# Longitudinal Associations Between Forgiveness and Psychological Flourishing in Chinese Early Adolescents: The Indirect Role of Psychological Resilience

**DOI:** 10.3390/bs16101716

**Published:** 2026-09-22

**Authors:** Lihui Zhang, Qiuyue Li, Yuanyuan Zhang, Juan Li

**Affiliations:** 1 Faculty of Education, Henan Normal University, Xinxiang 453007, China; 2410283140@stu.htu.edu.cn (Q.L.); 2410283166@stu.htu.edu.cn (Y.Z.); 2 School of Software, Henan Normal University, Xinxiang 453007, China; xl0067@htu.edu.cn

**Keywords:** forgiveness, psychological resilience, psychological flourishing, Chinese early adolescents

## Abstract

Positive psychological qualities are core protective resources for adolescents’ mental health and adaptive development. Although prior studies have confirmed the positive correlations among forgiveness, psychological resilience, and psychological flourishing, most empirical evidence is derived from cross-sectional data, leaving the longitudinal dynamics and potential indirect pathways among these constructs largely unexplored. To fill this research gap, this study explored the longitudinal associations among forgiveness, psychological resilience, and psychological flourishing in adolescents, with particular attention to the indirect role of psychological resilience. A two-wave longitudinal design spanning a six-month interval was adopted, involving 1034 Chinese early adolescents (47.49% male, n = 491; *M*_age_ = 13.29 years, *SD*_age_ = 0.75, age range was 12–15 years old at baseline), who completed self-report measures of forgiveness, psychological resilience, and psychological flourishing at both time points. Cross-lagged panel analyses revealed that T1 forgiveness significantly predicted T2 psychological flourishing, and T1 psychological flourishing significantly predicted T2 forgiveness. Further indirect effect testing indicated that psychological resilience showed significant indirect effects in both directions, whereas the direct effect was not significant. These findings suggest a bidirectional longitudinal relationship between forgiveness and psychological flourishing, with psychological resilience serving as a significant indirect pathway in both directions among Chinese early adolescents. This study provides empirical longitudinal evidence that may inform intervention efforts aimed at promoting adolescents’ sustainable psychological flourishing and mental health.

## 1. Introduction

One of the primary developmental tasks of adolescence is to cultivate positive adaptive patterns that enable effective navigation of complex interpersonal, academic, and self-identity challenges. In contrast to traditional paradigms focused on pathology alleviation, contemporary positive psychology emphasizes the promotion of psychological flourishing as a more holistic conception of mental health (Huppert & So, 2013; Seligman & Csikszentmihalyi, 2000; van Zyl et al., 2020). Psychological flourishing is characterized by high levels of subjective well-being and effective psychosocial functioning and is widely recognized as an important indicator of adolescent positive development (Waigel & Lemos, 2023).

Within the growing body of research on adolescent psychological flourishing, forgiveness has gained attention as a critical mechanism underlying positive psychological regulation. Forgiveness not only alleviates negative emotions and rumination but also plays a key role in psychological adjustment following adverse experiences (Wade et al., 2014; Vismaya et al., 2024; Q. Wang et al., 2018; L. Toussaint et al., 2023; McCullough, 2000). Moreover, as a positive psychological resource, it may be associated with recovery following major trauma and with positive psychological change (Gilo et al., 2022). It is positively correlated with multiple dimensions of well-being (Cowden et al., 2025); notably, its positive effects on subjective well-being and mental health are particularly evident among victims of bullying (Wulandari & Megawati, 2020). Prior research has relied predominantly on cross-sectional designs, making it difficult to draw conclusions about the direction of the relationship between forgiveness and psychological flourishing. While the broaden-and-build theory (Fredrickson, 2001) suggests positive affective states may enable forgiveness by mobilizing resources, this reverse pathway lacks direct empirical support. More significantly, understanding the reciprocal relationship between forgiveness and psychological flourishing is essential for revealing how individuals transition from post-traumatic growth to a state of psychological flourishing, and how this process may, in turn, deepen forgiveness. However, this question likewise remains an open question. To address this gap, the present study aims to examine the bidirectional predictive relationship between forgiveness and psychological flourishing and to further investigate whether psychological resilience accounts for indirect effects within this reciprocal relationship. In doing so, this study seeks to provide a more comprehensive understanding of the developmental processes linking forgiveness, psychological resilience, and psychological flourishing in adolescence.

## 2. Theoretical Framework and Hypothesis Development

### 2.1. Theoretical Framework

To clarify the bidirectional predictive relationship between forgiveness and psychological flourishing and to examine whether psychological resilience accounts for indirect effects within this reciprocal relationship, a theoretical framework is needed that can explain the dynamic interrelations among these constructs, particularly the role of psychological resources such as psychological resilience. This study therefore integrates broaden-and-build theory (Fredrickson, 2001) and the model of psychological resilience mechanisms (Davydov et al., 2010) as its core theoretical framework.

The broaden-and-build theory proposes that positive emotions may be accompanied by a broadening of an individual’s cognitive and behavioral repertoires and may be linked to the development of enduring psychological resources (e.g., psychological resilience, optimism), which may in turn be associated with future positive functioning (Fredrickson, 2001). The model of psychological resilience mechanisms, on the other hand, tends to conceptualize psychological resilience as a dynamic adaptive process rather than a fixed trait; it is thought to influence mental health through three potential pathways—buffering risks, facilitating recovery, and promoting growth (Davydov et al., 2010). Taken together, both theories appear to converge on a core proposition: positive psychological states may be associated with the development and consolidation of resources, and these resources may in turn be linked to adaptation and growth. Thus, forgiveness and psychological flourishing may be reciprocally associated, and psychological resilience may serve as an indirect pathway linking the two directions.

Building on these premises, this study employs a cross-lagged panel model (see Figure 1) in which forgiveness and psychological flourishing predict each other and psychological resilience serves as an indirect pathway in both directions. Specific hypotheses for each pathway are presented below.

### 2.2. The Relationship Between Adolescent Forgiveness and Psychological Flourishing

Psychological flourishing can be widely understood as an experience that combines good physical and mental health with a high level of happiness (L. Toussaint et al., 2022), and its connotation is further extended to aspects such as a sense of meaning, purpose, positive emotions, engagement, and achievement (Seligman, 2011). Psychological flourishing and forgiveness are closely related constructs, both reflecting core aspects of positive psychological functioning. Forgiveness refers to the internal psychological process in which an individual, after experiencing interpersonal offenses, actively dissolves resentment, relinquishes revenge, and may generate benevolence motivation (Barcaccia et al., 2020). It is further conceptualized as an emotion-focused coping response that may buffer or mediate the detrimental effects of unforgiveness on human psychological flourishing (L. Toussaint, 2022). In essence, both involve internal positive transformation, but whereas psychological flourishing seeks to cultivate the good, forgiveness advances well-being by diminishing the negative. Witvliet et al. (2022) observed that character strengths tend to cluster, providing a theoretical foundation for the bidirectional relationship examined here. That is, as relational strengths develop synergistically, forgiveness may foster psychological flourishing through interpersonal repair, while psychological flourishing, in turn, may endow individuals with greater resources to extend forgiveness. Numerous studies have shown that there may be a significant and positive bidirectional relationship between adolescent forgiveness and psychological flourishing.

Forgiveness, as an active psychological process, has multiple positive effects on the psychological flourishing of adolescents. Forgiveness alleviates anger, anxiety, depression, and stress, freeing up psychological resources that facilitate the generation of positive emotions, promote life satisfaction and subjective well-being (Rapp et al., 2022; Gao et al., 2022; Lavafpour Nouri et al., 2015; L. L. Toussaint et al., 2015), which lays the affective groundwork for psychological flourishing. In addition, it serves to buffer the adverse impact of stressful events (L. L. Toussaint et al., 2016) and helps resolve resentment and self-criticism (Skalski et al., 2024a). Research indicates that forgiveness is positively associated with adolescents’ self-acceptance, personal growth, and life satisfaction (Barcaccia et al., 2020; Pareek et al., 2016; Y. Zhang et al., 2024). Neuroimaging studies have revealed that forgiving engages brain regions implicated in meaning-making, suggesting that it facilitates the construction of positive meaning from interpersonal harm (Ricciardi et al., 2013; Schuttenberg et al., 2022). This body of research indicates that forgiveness functions not merely as a strategy for coping with hurt, but as an important resource that enables adolescents to alleviate psychological distress, construct meaning, achieve personal growth, and ultimately attain psychological flourishing (McCullough et al., 2003; Ho et al., 2024; Rapp et al., 2022; L. Toussaint, 2022). Accordingly, forgiveness may constitute an important pathway toward psychological flourishing. On this basis, we propose the following hypothesis:

**H1.**  *Forgiveness positively predicts subsequent psychological flourishing over time.*

Conversely, adolescents who experience psychological flourishing may also exhibit greater interpersonal tolerance. According to the broaden-and-build theory (Fredrickson, 2001), psychological flourishing may facilitate forgiving behavior by providing the necessary psychological resources (e.g., positive emotions and cognitive flexibility) while simultaneously reducing retaliatory motives. Cross-sectional evidence supports this notion; for instance, Ma and Jiang (2020) found that positive emotions (e.g., benevolence and prosocial affect) among adolescents indirectly enhance forgiveness through increased empathy. More compellingly, longitudinal studies have directly confirmed the predictive role of psychological flourishing. Prior subjective well-being and life satisfaction significantly predict individuals’ subsequent tendency to forgive (Bono et al., 2008; Y. Zhang et al., 2024). The aforementioned studies may suggest that psychological flourishing constitutes an important pathway toward forgiveness. On this basis, we propose the following hypothesis:

**H2.**  *Psychological flourishing positively predicts subsequent forgiveness levels across time.*

### 2.3. The Indirect Role of Psychological Resilience

Although previous studies have established a positive correlation between forgiveness and psychological flourishing (Yan et al., 2025; Mróz, 2023), the longitudinal interplay between these two constructs remains underexplored. Building on this empirical foundation, the present study further investigates whether psychological resilience accounts for indirect effects in this bidirectional relationship, representing the core focus of this research psychological resilience refers to an individual’s capacity to adapt effectively to adversity and maintain or restore healthy functioning (Fletcher & Sarkar, 2013). According to the model of psychological resilience mechanisms proposed by Davydov et al. (2010), psychological resilience influences mental health through three distinct pathways: protective mechanisms that buffer against risk factors, damage reduction and recovery mechanisms that facilitate a return to pre-trauma functioning, and facilitative mechanisms that promote post-traumatic growth and positive psychological experiences. This theoretical model provides a robust framework for examining the indirect role of psychological resilience in the present study.

The stress-and-coping model of forgiveness posits that forgiveness facilitates the adoption of adaptive coping strategies, such as problem-solving (Strelan, 2019), and reduces stressful reactions to transgressions, promoting health resilience and resolution in interpersonal relationships (Girgin Büyükbayraktar et al., 2025; Worthington & Scherer, 2004). These processes may indicate that forgiveness, as an adaptive coping strategy, could be closely linked to psychological resilience. Indeed, existing empirical research lends support to this inference: higher levels of forgiveness are associated with enhanced psychological resilience (Parveen & Pal, 2024; Worthington et al., 2016; Lestari & Huwae, 2023). Physiological evidence further supports this relationship: individuals with a greater propensity for forgiveness exhibit faster cardiovascular recovery following stress exposure, suggesting that forgiveness mitigates the sustained depletion of both physical and psychological resources (Larsen et al., 2012). Complementing these physiological findings, intervention-based evidence reveals that structured forgiveness training effectively promotes well-being, reduces anger, and bolsters psychological resilience (Akhtar & Barlow, 2018; Skalski et al., 2024a, 2024b; Biantoro & Hitipeuw, 2025). In turn, psychological resilience buffers the psychological damage stemming from various risk factors, enables individuals to recover from trauma, and facilitates post-traumatic growth as well as abundant positive psychological experiences (Davydov et al., 2010). Adolescents with higher levels of psychological resilience are better able to mobilize protective factors in their environment (e.g., social support) and employ positive coping strategies to regulate emotions (Yang et al., 2024). Such adaptive strengths could help adolescents pursue personal goals more effectively, alleviate negative affective states including anxiety and depression, and accumulate positive affective experiences and subjective well-being (Kaya & Odacı, 2024). All these positive outcomes contribute to individuals’ psychological flourishing, which refers to optimal psychological functioning that covers emotional, psychological, and social well-being, rather than merely the absence of mental illness (Bossert et al., 2024). Taken together, forgiveness may promote psychological flourishing by strengthening psychological resilience. Accordingly, we propose the following hypothesis:

**H3.**  *Psychological resilience would account for a significant indirect effect in the longitudinal association between prior forgiveness and subsequent psychological flourishing.*

However, this relationship may be bidirectional. Psychological flourishing, characterized by high levels of well-being and positive functioning, may in turn enhance psychological resilience. According to the Broaden-and-Build Theory (Fredrickson, 2001), positive emotions broaden cognitive and behavioral repertoires and build enduring resources, thereby improving individuals’ capacity to cope with adversity. Individuals who flourish psychologically are more likely to possess abundant psychological resources, which help sustain psychological resilience when facing stress. Longitudinal research by M. A. Thompson et al. (2021) found that positive emotions enhanced coping ability by building cognitive resources and could offset resource depletion caused by negative emotions. Sacro (2025) further verified, in a sample of emerging adults, that hope mediates the association between positive emotions and psychological resilience. Collectively, these empirical findings indicate that psychological flourishing can effectively enhance psychological resilience. At the same time, individuals with high psychological resilience are more likely to exhibit a tendency toward forgiveness, as they possess greater emotional regulation resources and more adaptive coping strategies (Agaibi & Wilson, 2005; Halilova et al., 2020; Yu & Liu, 2025). Prior research has demonstrated that higher psychological resilience is linked to fewer negative emotions, more positive emotions, and greater dispositional forgiveness (Cohn et al., 2009; Dwiwardani et al., 2014; Lozano et al., 2016; Seaton & Beaumont, 2014). Beyond these correlational findings, psychological resilience can serve as a positive predictor of forgiveness levels when individuals encounter specific interpersonal offenses (Halilova et al., 2020). Accordingly, we propose the following hypothesis:

**H4.**  *Psychological resilience would account for a significant indirect effect in the longitudinal association between prior psychological flourishing and subsequent forgiveness.*

The present study employed a cross-lagged panel design to explore the bidirectional relationship between adolescent forgiveness and psychological flourishing, as well as the longitudinal indirect effect of psychological resilience. The following research questions were addressed: (1) Do forgiveness and psychological flourishing mutually predict one another over time? (2) Does psychological resilience account for indirect effects in the longitudinal relationship between these two constructs? Figure 1 illustrates the hypothesized model.

## 3. Materials and Methods

### 3.1. Participants and Procedure

In this study, participants were recruited from a middle school in Xinxiang City, Henan Province, China. Two waves of questionnaire surveys were conducted with a six-month interval (Dormann & Griffin, 2015; Kwok et al., 2022; R. Zhang et al., 2023). A total of 1184 adolescents participated in the first survey (T1), and 1089 took part in the second survey (T2). After removing participants who failed the attention check items, the valid sample consisted of 1165 adolescents at T1 and 1057 at T2. Ultimately, only participants who completed both waves with valid data were retained for analysis, yielding a final sample of 1034 adolescents (47.49% male, n = 491), whose average age at the initial survey was 13.29 years (*SD* = 0.75), age range was 12–15 years old. Family characteristics, grade distribution, and age range are presented in Table 1.

Difference tests were performed to examine the differences between adolescents who participated at all time points and those who dropped out at T2. The results revealed that there were no significant differences between the two groups at T1 in forgiveness (*M*_dropout_ = 62.10, *SD*_dropout_ = 13.06, *M*_stay_ = 62.81, *SD*_stay_ = 12.32; *t*(1163) = 0.62, *p* = 0.54), psychological resilience (*M*_dropout_ = 88.22, *SD*_dropout_ = 18.10, *M*_stay_ = 89.46, *SD*_stay_ = 17.38; *t*(1163) = 0.77, *p* = 0.45), and psychological flourishing (*M*_dropout_ = 37.56, *SD*_dropout_ = 9.70, *M*_stay_ = 39.18, *SD*_stay_ = 9.22; *t*(1163) = 1.879, *p* = 0.06). This indicates that there is no structured attrition of participants across the two time points.

This study was approved by the ethics committee of the institution to which the first author belongs and was conducted in accordance with the Declaration of Helsinki and its later amendments. The survey was conducted with prior approval from the participating schools. After school approval was obtained, written informed consent was obtained from the parents/guardians of all participants, and assent was obtained from the adolescents themselves, before the questionnaire was administered. All participants and their parents/guardians were clearly informed that all data would be de-identified and that strict confidentiality would be guaranteed; participation was completely voluntary, and participants could withdraw at any time. Data collection was supervised by trained graduate students who followed standardized instructions throughout, and the survey was conducted entirely in classrooms using paper-and-pencil questionnaires. Volunteers received compensation (e.g., stationery) after the study.

### 3.2. Measures

#### 3.2.1. Forgiveness

This study used the Heartland Forgiveness Scale (HFS), which was developed by L. Y. Thompson et al. (2005) and translated into Chinese by H. Zhang (2009). The scale includes three subscales: forgiveness of self, forgiveness of others, and forgiveness of situations, with a total of 14 items. It uses a 7-point Likert scale. Higher scores indicate a greater tendency to forgive. The Chinese version of the Heartland Forgiveness Scale, which has been validated among Chinese samples with good validity and reliability, was used in this study (Yin et al., 2026). In the present study, Cronbach’s α coefficients of this scale at T1 and T2 were 0.810 and 0.833, respectively.

#### 3.2.2. Psychological Resilience

The scale used in this study was developed by Y. Q. Hu and Gan (2008) based on the psychological resilience process model. Within this scale, psychological resilience is divided into two major parts: personal strength and support. Personal strength comprises three dimensions: goal focus, emotional regulation, and positive cognition. Support includes two dimensions: family support and interpersonal support. The scale contains 27 items, rated on a 5-point Likert scale. Higher scores indicate stronger psychological resilience. In the present study, Cronbach’s α coefficients for this scale at T1 and T2 were 0.895 and 0.904, respectively.

#### 3.2.3. Psychological Flourishing

This study used the Flourishing Scale to measure adolescents’ psychological flourishing (Diener et al., 2010). The questionnaire is unidimensional, consisting of 8 items, and employs a 7-point Likert scale. There are no reverse-scored items. The scale mainly assesses eight important aspects of human positive functioning: positive relationships, self-acceptance, competence, meaning and purpose, optimism, engagement, being respected, and contributing to the well-being of others. Higher total scores indicate higher levels of psychological flourishing. The Chinese version of this scale has been validated and demonstrates satisfactory reliability and validity among Chinese adolescents (Duan & Xie, 2019; X. Tang et al., 2016). In this study, Cronbach’s α coefficients for this scale at T1 and T2 were 0.897 and 0.913, respectively.

### 3.3. Data Analyses

First, confirmatory factor analysis was conducted using Mplus 8.30 to test longitudinal measurement invariance for adolescent forgiveness, psychological resilience, and psychological flourishing. A series of nested models were tested sequentially: a configural invariance model (baseline), a weak invariance model with factor loadings constrained to be equal across time, and a strong invariance model with both factor loadings and intercepts constrained equal across time. Measurement invariance was evaluated based on changes in CFI (ΔCFI) and RMSEA (ΔRMSEA), with ΔCFI < 0.01 and ΔRMSEA < 0.015 indicating that longitudinal measurement invariance was established (Chen, 2007). Next, correlations among variables were analyzed using SPSS 20.0. Subsequently, a cross-lagged panel model was constructed to examine the bidirectional relationship between forgiveness and psychological flourishing. Finally, a cross-lagged indirect effects model incorporating forgiveness, psychological resilience, and psychological flourishing was tested. Notably, for measurement invariance testing, latent-variable CFA was conducted with forgiveness, psychological resilience, and psychological flourishing specified as first-order latent factors. For the subsequent cross-lagged panel and two-wave indirect-effect analysis, total scores of each scale were used as observed variables. This distinction is clearly stated to ensure transparency in our modeling strategy. To rule out potential confounding effects, gender and age were included as covariates in both the cross-lagged panel model and the indirect effect testing, ensuring that all estimated paths were adjusted for these demographic factors. The final analytic sample consisted of adolescents who completed both waves; therefore, no wave-level missingness remained. A small number of participants had missing responses on a few individual items (item-level missingness). Full information maximum likelihood (FIML) estimation in Mplus 8.30 was used to handle this item-level missingness. All statistical tests were two-tailed, with *p* < 0.05 considered statistically significant.

## 4. Results

To empirically test the four proposed hypotheses, the results are presented in accordance with the analytical strategy delineated in the Method section. The exposition proceeds through a series of requisite preliminary diagnostics, followed by the primary hypothesis-testing analyses. Specifically, we first report the common method bias test to rule out the possibility that systematic variance may distort the observed relationships. We then present descriptive statistics and bivariate correlations among the key variables, which provide preliminary evidence for the anticipated associations. Next, we detail the measurement invariance tests across time points, a necessary prerequisite for drawing valid inferences from longitudinal comparisons. Subsequently, we perform the cross-lagged panel model analysis to directly examine the reciprocal and directional relationships posited in the hypotheses (Hypotheses 1 and 2). Finally, we test the longitudinal indirect pathway to evaluate the indirect effects and temporal sequencing specified in the theoretical model (Hypotheses 3 and 4). This stepwise progression ensures that each analytical phase logically builds upon the preceding one, culminating in a comprehensive empirical evaluation of the proposed theoretical framework.

### 4.1. Common Method Bias Test

To address common method bias, procedural and statistical approaches were employed. Procedurally, data collection at two time points helped mitigate potential bias. Statistically, Harman’s single-factor test showed that the first unrotated factor accounted for 26.32% of the total variance. This result provides some initial evidence that common method variance is not a serious concern in this study. However, given both the low sensitivity of Harman’s test as a screening tool (Podsakoff et al., 2012; D. Tang & Wen, 2020) and the fact that all constructs were measured via adolescent self-report, the possibility of common method variance cannot be completely excluded and should be considered when interpreting the findings.

### 4.2. Descriptive Statistics and Correlation Analysis

Descriptive statistical analysis and Pearson correlation analysis were conducted to examine the variables of interest. The results showed that forgiveness, psychological resilience, and psychological flourishing were positively and significantly intercorrelated both within each time point and across the two time points. Gender was negatively and significantly correlated with each of these variables at both time points, whereas age was not significantly correlated with any of them. See Table 2 for detailed correlation coefficients.

### 4.3. Measurement Invariance Test

Given that data were collected at two time points, establishing longitudinal measurement invariance was necessary to ensure the stability and reliability of the cross-lagged analyses. As shown in Table 3, following the criteria recommended by Chen (2007), ΔCFI < 0.01 and ΔRMSEA < 0.015 were adopted as thresholds for accepting measurement invariance. The results indicated that the configural, metric, and scalar invariance models for each scale met these criteria, confirming that longitudinal measurement invariance was established for all three measures. Consequently, scalar invariance constraints were retained in subsequent analyses.

### 4.4. Cross-Lagged Panel Model Analysis

This study examined the longitudinal relationship between forgiveness and psychological flourishing through cross-lagged panel analysis, with gender and age included as control variables. The model was estimated using total scores for each construct, with all possible regression paths (i.e., autoregressive paths, cross-lagged paths, and contemporaneous correlations) freely estimated. The model was just-identified (saturated) with zero degrees of freedom, yielding the following fit indices: *χ*^2^(0) = 0, CFI = 1, TLI = 1, RMSEA = 0, and SRMR = 0. Parameter estimates are presented in Figure 2; both constructs demonstrated substantial temporal stability. Specifically, T1 forgiveness had a significant positive prospective association with T2 forgiveness (*β* = 0.59, *SE* = 0.03, *p* < 0.001), and T1 psychological flourishing had a significant positive prospective association with T2 psychological flourishing (*β* = 0.61, *SE* = 0.02, *p* < 0.001). Regarding cross-lagged effects, T1 forgiveness significantly predicted T2 psychological flourishing (*β* = 0.13, *SE* = 0.03, *p* < 0.001), indicating that initial levels of forgiveness is a prospective predictor of subsequent psychological flourishing. Conversely, T1 psychological flourishing significantly predicted T2 forgiveness (*β* = 0.10, *SE* = 0.03, *p* = 0.001), suggesting that early psychological flourishing also prospectively predicted later forgiveness. These findings provide evidence for a bidirectional predictive relationship between forgiveness and psychological flourishing.

### 4.5. Two Wave Indirect Effect Analysis of Psychological Resilience

Following the procedure for two wave indirect effect analysis proposed by Fang et al. (2021), we specified a first-order cross-lagged panel model (CLPM) to examine the indirect role of psychological resilience in the association between forgiveness and psychological flourishing, with gender and age included as control variables. Two indirect effects were computed: (a) the product of the cross-lagged path from T1 Forgiveness to T2 Psychological resilience (a1 path) and the cross-lagged path from T1 Psychological resilience to T2 Psychological flourishing (b1 path), and (b) the product of the cross-lagged path from T1 Psychological flourishing to T2 Psychological resilience (a2 path) and the cross-lagged path from T1 Psychological resilience to T2 Forgiveness (b2 path). Bias-corrected bootstrap confidence intervals with 5000 resamples were used to test the indirect effects. This approach has been used in prior longitudinal studies to examine whether a predictor is indirectly associated with an outcome through a variable assessed at a given time point (Li et al., 2024; X. C. Wang et al., 2023). The model was estimated using total scores for each construct, with all possible regression paths (i.e., autoregressive paths, cross-lagged paths, and contemporaneous correlations) freely estimated. The model was just-identified (saturated) with zero degrees of freedom, yielding the following fit indices: *χ*^2^(0) = 0, CFI = 1, TLI = 1, RMSEA = 0, and SRMR = 0.

As shown in Table 4 and Figure 3, after controlling for the autoregressive paths of T1 Forgiveness on T2 Forgiveness (*β* = 0.54, *p* < 0.001), T1 psychological resilience on T2 psychological resilience (*β* = 0.46, *p* < 0.001) and the stability effect of T1 psychological flourishing on T2 psychological flourishing (*β* = 0.51, *p* < 0.001), structural equation modeling analysis showed T1 forgiveness significantly positively predicted T2 psychological resilience (*β* = 0.14, *SE* = 0.03, *p* < 0.001). Furthermore, T1 psychological resilience significantly positively predicted T2 psychological flourishing (*β* = 0.20, *SE* = 0.04, *p* < 0.001). Psychological resilience accounted for a significant indirect effect in the longitudinal association from Time 1 forgiveness to Time 2 psychological flourishing (a1 × b1 = 0.03, 95% CI = [0.02, 0.04]). Furthermore, T1 psychological resilience significantly positively predicted T2 forgiveness (*β* = 0.12, *SE* = 0.04, *p* = 0.005). Furthermore, T1 psychological flourishing significantly positively predicted T2 psychological resilience (*β* = 0.21, *SE* = 0.03, *p* < 0.001). Psychological resilience accounted for a significant indirect effect in the prospective association from T1 psychological flourishing to T2 forgiveness (a2 × b2 = 0.03, 95% CI = [0.01, 0.04]). At the same time, after adding T1 psychological resilience and T2 psychological resilience to the model, the direct paths from T1 psychological flourishing to T2 forgiveness (*β* = 0.05, SE = 0.04, *p* = 0.227) and from T1 forgiveness to T2 psychological flourishing (*β* = 0.05, SE = 0.03, *p* = 0.098) were both non-significance.

Given that the indirect-effect model comprises only two measurement occasions, it cannot establish a conventional temporal sequence of indirect effects. Accordingly, the indirect effect should be interpreted as a prospective indirect association rather than a causal mediation effect; the results should therefore be interpreted with caution.

## 5. Discussion

This study employed a two-wave longitudinal design to examine the longitudinal relationships among forgiveness, psychological resilience, and psychological flourishing in Chinese adolescents. Cross-lagged analyses were first performed to test the bidirectional prospective associations between forgiveness and psychological flourishing, followed by an examination of the indirect effects of psychological resilience in this reciprocal process. Compared with cross-sectional designs, the two-wave design allows for the establishment of temporal precedence and the detection of prospective associations, though it does not permit causal inferences. However, the traditional cross-lagged panel model (CLPM) used in this study, with only two waves of data, cannot disentangle between-person differences from within-person changes. Accordingly, the observed cross-lagged estimates may reflect a mixture of between-person and within-person sources of variance and should not be interpreted as pure within-person dynamic effects. Readers should bear these boundary conditions in mind when interpreting the subsequent results.

### 5.1. The Relationship Between Adolescent Forgiveness and Psychological Flourishing

The cross-lagged panel analysis revealed significant autoregressive effects for both forgiveness and psychological flourishing, suggesting considerable stability in these two constructs across early adolescence. In addition, T1 forgiveness positively predicted subsequent T2 psychological flourishing, and T1 psychological flourishing also positively predicted subsequent T2 forgiveness. These results confirmed a reciprocal, bidirectional association between forgiveness and psychological flourishing, consistent with the study hypotheses. Forgiveness may facilitate psychological flourishing by conserving mental resources through the alleviation of interpersonal stress, reduction in negative emotional rumination, and promotion of relationship repair (Parveen & Pal, 2024; Raj et al., 2016; Worthington & Scherer, 2004). During adolescence, when peer relationships become increasingly salient, forgiveness enables teenagers to resolve interpersonal conflicts and maintain mutually supportive relationships (Flanagan et al., 2012; Komesuor et al., 2023; Girgin Büyükbayraktar et al., 2025), thereby providing a crucial social resource foundation for the long-term development of adolescents’ psychological flourishing. In addition, this study further demonstrated that psychological flourishing positively predicts subsequent changes in adolescents’ forgiveness, confirming a reciprocal longitudinal association between the two variables. Positive emotions and psychological flourishing may support adolescents’ cognitive flexibility, emotion regulation capacities, and motivation for social connection, which could making them more likely to adopt constructive responses, such as forgiveness, when confronted with interpersonal transgressions (Morrish et al., 2018; Komesuor et al., 2023). These reciprocal prospective associations suggest that forgiveness and psychological flourishing may be linked in a mutually reinforcing pattern among early adolescents, and that this pattern may be associated with better psychosocial adjustment.

In addition, it should be noted that, after psychological resilience was included in the cross-lagged model, the direct cross-lagged paths from forgiveness to psychological flourishing and from psychological flourishing to forgiveness were no longer significant. Contrary to our initial hypotheses, after psychological resilience was included in the cross-lagged model, the direct cross-lagged paths from forgiveness to psychological flourishing and from psychological flourishing to forgiveness were no longer significant. A conceptual feature of coping strategies is that coping with stress and adversity involves the mobilization of specific psychological resources to restore or maintain functioning. Within the present theoretical framework, forgiveness as a positive coping strategy would be expected to show an association with psychological flourishing that is intertwined with an indirect pathway involving psychological resilience, rather than appearing as an independent direct path. However, given the two-wave design, these results should be interpreted with caution, and causal mediation cannot be established. We therefore describe the findings as evidence of significant indirect pathways with nonsignificant direct paths within the specified model, rather than using terms such as “full mediation”.

### 5.2. The Indirect Role of Psychological Resilience

This study also found that psychological resilience played an indirect role in the bidirectional relationship between forgiveness and psychological flourishing in adolescents, a result that supported Hypothesis 3 and Hypothesis 4. This suggests that psychological resilience significantly accounted for the reciprocal longitudinal association observed between forgiveness and psychological flourishing.

This study extends the mechanistic model of psychological resilience proposed by Davydov et al. (2010) by examining its application to the link between forgiveness and psychological flourishing among adolescents. When adolescents who have experienced interpersonal harm engage in forgiveness, the protective mechanism of psychological resilience comes into play by promoting adaptive emotion regulation (Gross, 2015), thereby buffering the impact of reactive emotions like resentment and anger and sustaining immediate psychological stability (J. Hu et al., 2025). Following the decision to forgive, the recovery mechanism is activated, manifesting as the individuals’ use of positive emotions to accelerate recovery from psychological distress (Tugade & Fredrickson, 2004) and reduce rumination regarding hurtful interpersonal events (Chatzimike-Levidi & Collard, 2023). Ultimately, the facilitation mechanism enables deeper meaning-making and cognitive integration. Empirical evidence confirms that this process constitutes a central pathway to post-traumatic growth and fosters the development of positive qualities (Elam & Taku, 2022; García-Vázquez et al., 2020). These psychological resilience-fostered positive changes align with and promote multiple dimensions of psychological flourishing (Ahmed, 2024), including positive emotional experiences, satisfying relationships, clear life goals, and continued personal growth (Seligman, 2011). Furthermore, psychological flourishing may also prospectively predict forgiveness, with psychological resilience serving as an indirect pathway in this direction. The positive emotions and sense of meaning inherent in psychological flourishing broaden individuals’ cognitive resources (Fredrickson, 2001), strengthen their psychological resilience against adversity, and ultimately promote the development of psychological resilience. Individuals with higher psychological resilience possess superior emotional regulatory resources and adaptive coping skills, which enable them to readily forgive others (Agaibi & Wilson, 2005; Halilova et al., 2020), themselves, and even adverse environmental circumstances.

It should be noted that the theoretical framework adopted in this study, particularly the model of psychological resilience mechanisms (Davydov et al., 2010), conceptualizes psychological resilience as a dynamic adaptive process rather than a fixed trait. In the present study, however, psychological resilience was defined as an individual’s capacity to adapt effectively to adversity and maintain or restore healthy functioning (Fletcher & Sarkar, 2013), a definition consistent with the multidimensional self-report measure used, which captures goal focus, emotional regulation, positive cognition, family support, and interpersonal support. This measure reflects self-reported capacities and resources rather than directly observed dynamic processes. Accordingly, the mechanistic interpretations discussed above, drawn from the model of psychological resilience mechanisms, should be understood as theoretically plausible explanations of the observed associations rather than as processes directly demonstrated by the present analyses.

### 5.3. Limitations and Future Directions

Although this study provided longitudinal evidence for understanding the relationships among adolescent forgiveness, psychological resilience, and psychological flourishing, several limitations should be acknowledged and addressed in future research. First, this study employed a two-wave longitudinal design, which allowed for preliminary examination of predictive relationships among variables. However, with data collected at only two time points, the explanatory power regarding how psychological resilience functions as a longitudinal mediator between forgiveness and psychological flourishing remained limited. Accordingly, we limited the interpretation of the findings to the two-wave indirect effects. Moreover, although the traditional cross-lagged panel model (CLPM) controlled for autoregressive effects and established temporal precedence, its inherent limitation lies in conflating between-person differences with within-person changes, such that the observed cross-lagged estimates may reflect a mixture of between-person and within-person sources of variance and should not be interpreted as pure within-person dynamic effects. In contrast, the random-intercept cross-lagged panel model (RI-CLPM) can disentangle stable between-person variance from fluctuating within-person variation, thereby providing more rigorous parameter estimates. However, the identification and estimation of this model require data from at least three time points. Accordingly, the present findings should be interpreted as evidence of prospective associations rather than as confirmation of causal effects. Future research is advised to adopt three or more waves of longitudinal data and to incorporate RI-CLPM analyses, so as to more purely examine the dynamic mediating role of psychological resilience at the within-person level in the relationship between forgiveness and psychological flourishing.

Second, all variables in this study were measured via self-report. Although we employed Harman’s single-factor test to statistically examine common method bias, and the results provided initial evidence that common method variance did not pose a serious concern in this study, the possibility of common method bias cannot be completely ruled out. This is due to both the low sensitivity of Harman’s test as a screening tool (Podsakoff et al., 2012; D. Tang & Wen, 2020) and the fact that all constructs were assessed through adolescent self-report. Accordingly, this limitation should be taken into consideration when interpreting the present findings. Future research is encouraged to adopt multi-source data collection strategies (e.g., peer reports, teacher evaluations, or behavioral indicators), or to temporally separate the measurement of predictors and outcome variables, so as to more effectively control for common method bias at the design level and enhance the robustness of the conclusions.

Third, the sample in this study was drawn from a single middle school in Henan Province, which limited the generalizability of the findings to adolescents from different regions, cultural backgrounds, or socioeconomic statuses. Future research should replicate these findings with more diverse and representative samples.

In addition, while this study examined intrinsic psychological factors, it did not account for key environmental and situational variables (e.g., family environment, stressful life events) that may have jointly affected forgiveness, psychological resilience, and psychological flourishing. Future studies should include these factors as covariates to enhance model precision.

Finally, although FIML was employed under the MAR assumption, this assumption could not be empirically verified. Attrition analyses showed no significant baseline differences between retained and lost participants on key baseline variables, reducing concern about systematic attrition. However, these analyses do not establish the MAR assumption or rule out the possibility of MNAR. Therefore, the possibility of nonrandom missingness cannot be excluded, and the findings should be interpreted with caution.

### 5.4. Implications

At the theoretical level, this study contributes in two key ways. First, by identifying psychological resilience as a significant indirect pathway in the bidirectional relationship between forgiveness and psychological flourishing, this research provides longitudinal evidence in support of the broaden-and-build theory. Previous applications of this framework have predominantly focused on how positive emotions broaden cognitive resources and build enduring personal resources. By demonstrating that forgiveness and psychological flourishing are linked through psychological resilience as a bidirectional indirect pathway, the present study offers preliminary longitudinal evidence for the dynamic upward spiral posited by the broaden-and-build theory. Second, this study extends the literature on psychological resilience resources. Although psychological resilience has been widely recognized as a critical protective factor in adolescent development, its mediating role within positive developmental cycles remains insufficiently examined. The finding that psychological resilience constitutes a significant indirect pathway in both directions suggests that psychological resilience may play a pivotal role in the reciprocal interplay between forgiveness and psychological flourishing, thereby providing novel empirical insight into the functional role of psychological resilience in positive developmental processes.

At the practical level, the findings yield preliminary implications for the design of intervention programs. First, the significant indirect pathway via psychological resilience suggests that psychological resilience may represent a viable target for future intervention research. School-based programs aimed at promoting adolescent mental health and positive development could consider integrating psychological resilience-enhancing components—such as cognitive reappraisal training, emotion regulation skills, and social support strengthening—into broader social–emotional learning curricula. However, given that the present study did not experimentally manipulate psychological resilience, it remains an open empirical question whether enhancing psychological resilience would produce causal effects on positive developmental outcomes. Accordingly, definitive intervention recommendations await rigorous validation through experimental designs, including randomized controlled trials, before such implications can be translated into practice. Second, the bidirectional pattern identified in this study implies that interventions targeting either forgiveness or psychological flourishing may yield mutually reinforcing effects. This hypothesis could be tested in future experimental studies by comparing the efficacy of single-component interventions (targeting forgiveness or psychological flourishing alone) against combined interventions (incorporating both forgiveness and psychological resilience components) to determine whether synergistic effects emerge. Importantly, these implications should be interpreted as hypothesis-generating rather than prescriptive in nature. Future research may draw upon these suggestions as a foundation for designing experimental studies that rigorously examine whether and how forgiveness- and psychological resilience-based interventions facilitate adolescent growth and development. Practitioners and policymakers are encouraged to treat these findings as preliminary evidence to inform the planning of future pilot programs, with the understanding that efficacy evaluation constitutes a necessary subsequent step.

## 6. Conclusions

This study drew the following conclusions: (1) forgiveness and psychological flourishing were reciprocally and prospectively associated across the two waves among Chinese early adolescents, and (2) psychological resilience was significantly and indirectly associated with both directions of these reciprocal prospective associations. These findings suggest reciprocal prospective associations between forgiveness and psychological flourishing, with psychological resilience serving as a significant indirect pathway in both directions. However, given the two-wave correlational design, these findings should be interpreted as associations rather than causal effects. In addition, because all participants were recruited from one middle school in Henan Province, the conclusions should be restricted to early adolescents similar to those in the present sample rather than to Chinese adolescents more broadly. The findings provide longitudinal evidence that may inform future research and intervention efforts targeting psychological resilience, although intervention effects remain to be tested.

## Figures and Tables

**Figure 1:**
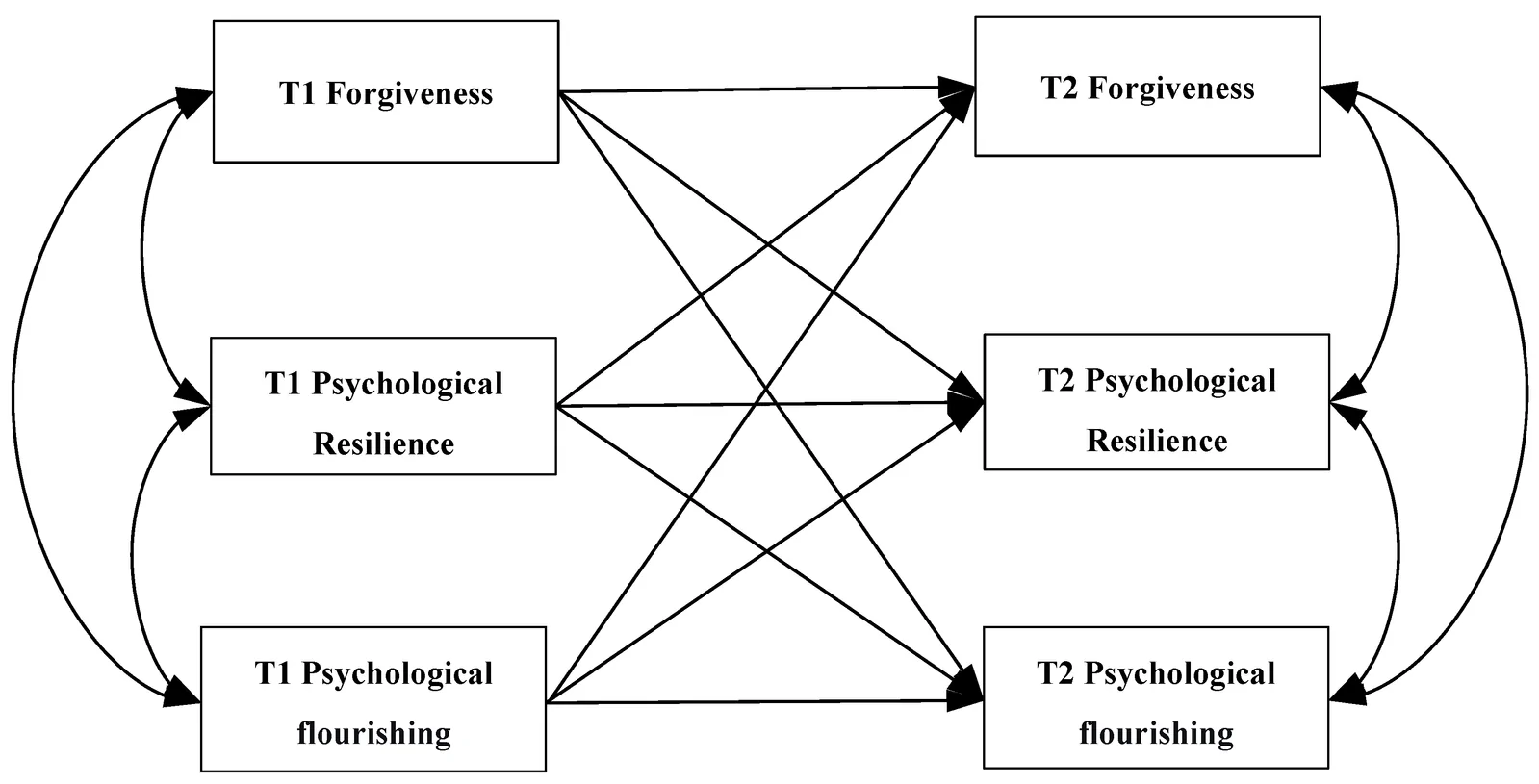
A Cross-Lagged Panel Model (CLPM) of Forgiveness, Psychological Resilience, and Psychological Flourishing Among Chinese Early Adolescents.

**Figure 2:**
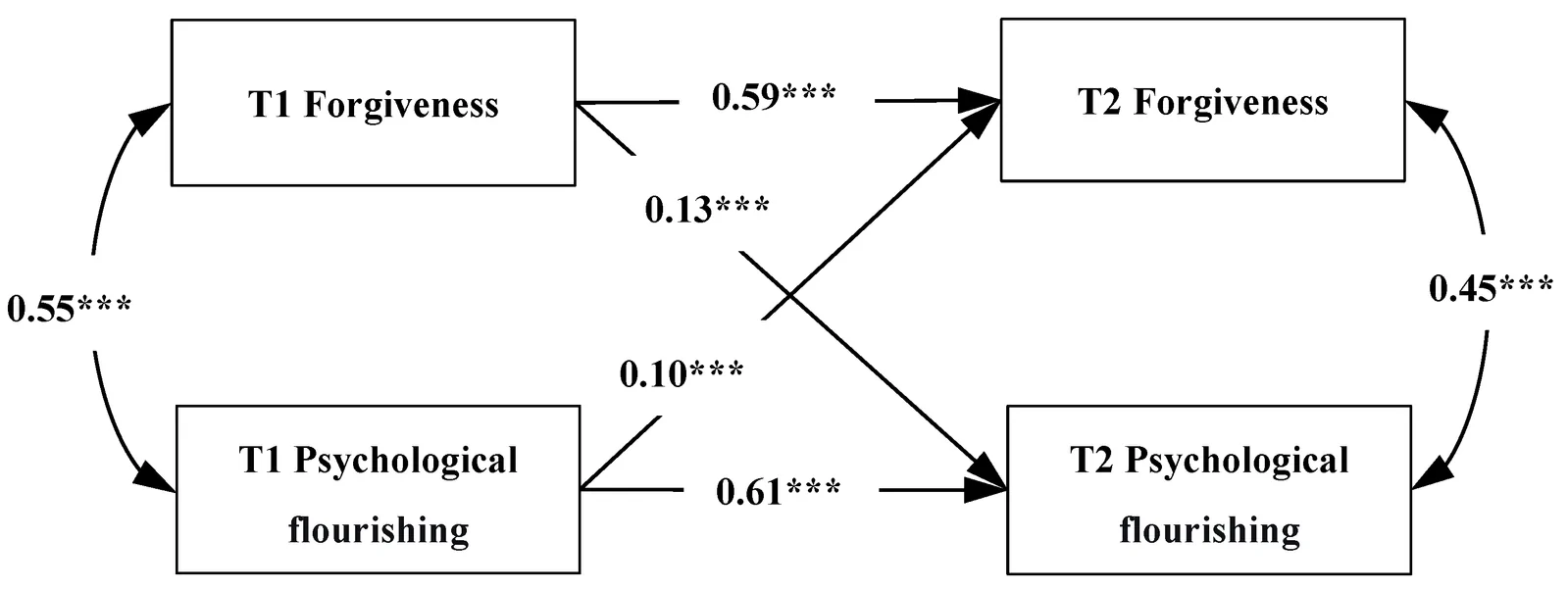
Cross-lagged Effects of Forgiveness and Psychological Flourishing Among Chinese Early Adolescents. *** *p* < 0.001.

**Figure 3:**
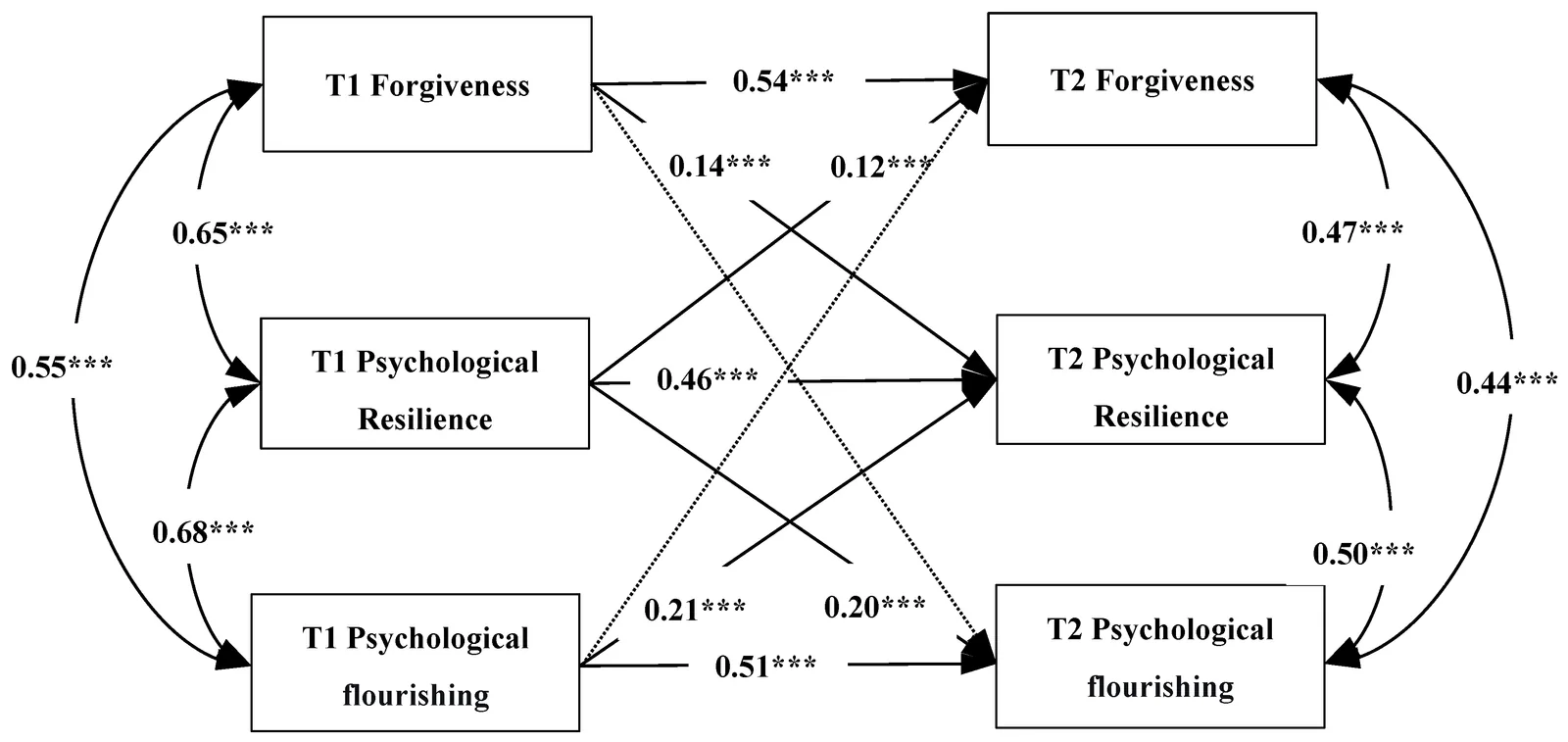
Longitudinal Indirect Effect of Psychological Resilience Among Chinese Early Adolescents. *** *p* < 0.001.

**Table 1:** Demographic information of the sample.

Demographic Information	Level	Percentage
Age		
	12 years	15.09%
	13 years	42.55%
	14 years	38.97%
	15 years	2.42%
	Not reported	0.97%
Grade		
	7th grade	49.2%
	8th grade	50.8%
Only child		
	Yes	16.0%
	No	83.7%
	Not reported	0.3%
Parental migrant work status	Both parents working away from home	4.2%
	One parent working away from home	26.9%
	Neither parent working away from home	68.5%
	Not reported	0.5%

**Table 2:** Descriptive statistical analysis and correlation analysis of each variable (*N* = 1034).

Variables	*M*	*SD*	1	2	3	4	5	6	7
Gender	1.53	0.50	1						
Age	13.28	0.75	−0.10 **	1					
T1 Forgiveness	62.80	12.32	−0.21 ***	−0.01	1				
T1 Psychological resilience	89.46	17.38	−0.17 ***	0.02	0.66 ***	1			
T1 Psychological flourishing	39.18	9.22	−0.11 ***	0.02	0.56 ***	0.69 ***	1		
T2 Forgiveness	62.18	12.52	−0.22 ***	0.04	0.66 ***	0.52 ***	0.43 ***	1	
T2 Psychological resilience	89.05	17.10	−0.18 ***	0.05	0.56 ***	0.70 ***	0.61 ***	0.67 ***	1
T2 Psychological flourishing	39.10	9.36	−0.11 ***	0.02	0.47 ***	0.59 ***	0.68 ***	0.60 ***	0.72 ***

Note: ** *p* < 0.01, *** *p* < 0.001.

**Table 3:** Longitudinal Measurement Invariance Test of Study Variables.

Variable	Model	*χ* ^2^	*df*	CFI	TLI	RMSEA	Model Comparison	ΔCFI	ΔRMSEA
Forgiveness	M1	784.17	134	0.928	0.902	0.069			
	M2	803.20	145	0.927	0.908	0.066	M2 vs. M1	0.001	0.003
	M3	839.88	156	0.924	0.911	0.065	M3 vs. M2	0.003	0.001
Psychological resilience	M1	2371.67	624	0.915	0.904	0.052			
	M2	2401.30	646	0.914	0.907	0.051	M2 vs. M1	0.001	0.001
	M3	2426.62	668	0.914	0.910	0.050	M3 vs. M2	0	0.001
Psychological flourishing	M1	294.98	36	0.971	0.956	0.083			
	M2	309.67	43	0.971	0.962	0.077	M2 vs. M1	0	0.006
	M3	325.52	50	0.970	0.966	0.073	M3 vs. M2	0.001	0.004

**Table 4:** Path Estimates and Indirect Effects in the Cross-Lagged Mediation Model.

	Estimate		SE	LLCI	ULCI	*p*
	B	*β*				
Autoregressive path						
T1 Forgiveness to T2 Forgiveness	0.55	0.54	0.03	0.48	0.61	<0.001
T1 Psychological resilience to T2 Psychological resilience	0.45	0.46	0.04	0.38	0.53	<0.001
T1 Psychological flourishing to T2 Psychological flourishing	0.52	0.51	0.04	0.44	0.59	<0.001
Cross-lagged path 1						
T1 Forgiveness to T2 Psychological resilience (a1)	0.19	0.14	0.03	0.08	0.20	<0.001
T1 Psychological resilience to T2 Psychological flourishing (b1)	0.11	0.20	0.04	0.12	0.28	<0.001
T1 Forgiveness to T2 Psychological flourishing (c1)	0.04	0.05	0.03	−0.01	0.11	0.098
Indirect effect (a1 × b1)	0.03		0.01	0.02	0.04	<0.001
Cross-lagged path 2						
T1 Psychological flourishing to T2 Psychological resilience (a2)	0.39	0.21	0.03	0.15	0.28	<0.001
T1 Psychological resilience to T2 Forgiveness (b2)	0.08	0.12	0.04	0.03	0.20	0.005
T1 Psychological flourishing to T2 Forgiveness (c2)	0.06	0.05	0.04	−0.03	0.11	0.227
Indirect effect (a2 × b2)	0.03		0.01	0.01	0.04	0.005

## Data Availability

The data presented in this study are available on request from the corresponding author due to ethical restrictions (the need to protect participant confidentiality and comply with institutional review board policies).
